# Double-opposing negative pressure dressing—a useful method for splinting skin grafts on the penile shaft

**DOI:** 10.1093/jscr/rjae529

**Published:** 2024-08-23

**Authors:** Steven L Zhang, Benjamin Z W Chung, Allen Wei-Jiat Wong

**Affiliations:** Section of Plastic, Reconstructive and Aesthetic Surgery, Department of General Surgery, Woodlands Health, 17 Woodlands Dr 17, Singapore 737628, Singapore; Section of Plastic, Reconstructive and Aesthetic Surgery, Department of General Surgery, Tan Tock Seng Hospital, 11 Jln Tan Tock Seng, Singapore 308433, Singapore; Plastic, Reconstructive & Aesthetic Surgery Service, Sengkang General Hospital, 110 Sengkang E Way, Singapore 544886, Singapore

**Keywords:** plastic surgery, reconstructive surgery, penile reconstruction, skin graft, negative pressure dressing, wound care

## Abstract

The care of skin grafts in the penile shaft is challenging because of its cylindrical shape and constantly changing length and lie, which makes it difficult to apply uniform compression and ensure immobilization during the critical period of skin graft take. These challenges are difficult to overcome with conventional dressings. The authors describe a technique of applying a double-opposing negative pressure dressing to sandwich the penile shaft following reconstruction with a skin graft, which is simple to apply and addresses these issues. Adoption of this technique may allow the reconstructive surgeon to manage skin grafts on the penile shaft with greater ease and confidence of optimum graft take.

## Introduction

The penile shaft often requires reconstruction with a skin graft following tissue loss for various reasons including malignancy, infection, trauma, burns, and lymphedema [1]. The application of dressings in this region is often challenging because of the cylindrical shape of the penile shaft and constantly changing length and lie, which makes it difficult to apply uniform compression and ensure immobilization during the critical period of skin graft take. As a result, there is often a variable degree of graft loss to be expected, especially over the dorsum and base of penis [1]. Conventionally, a variety of dressings have been used in an effort to optimize compression and immobilization, including tie-over bolsters [2], foam bolsters [3–5], modified syringe splints [6], and plaster splints [2]. Even with such adjuncts, however, it is still difficult to achieve adequate immobilization and maintenance of penile shaft stretch. The authors describe their technique of applying a double-opposing negative pressure dressing to address these issues.

## Case report

A 45-year-old male patient with a past medical history of hypertension was referred to the Plastic Surgery service for penile skin reconstruction. He had biopsy-proven Extramammary Paget’s disease (EMPD) of the penile shaft and base, and had undergone a mapping biopsy prior to planned wide excision [7]. He was counselled for and underwent wide excision of the EMPD and reconstruction with a split thickness skin graft and local advancement flaps.

**Figure 1:**
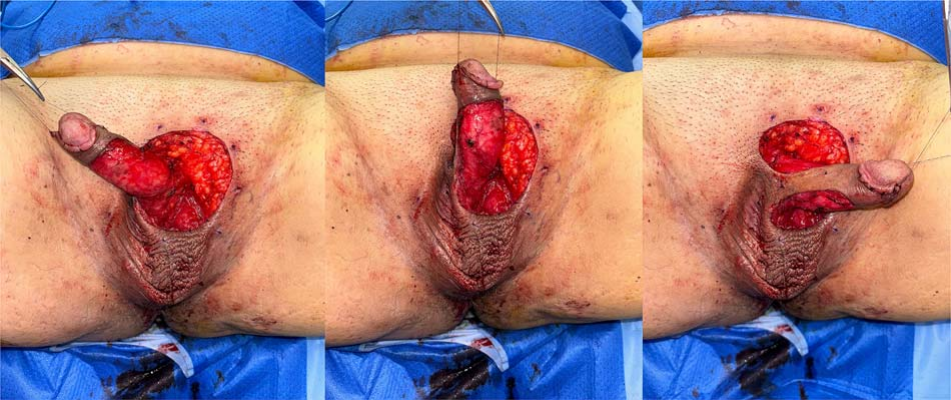
Near circumferential defect of the penile shaft skin following wide excision of EMPD.

Following wide excision, there was a near-circumferential defect of the penile shaft and base (Fig. 1). Mons and scrotal advancement flaps were elevated to resurface the penile base. A split thickness skin graft was harvested from the left thigh with a dermatome at 12/1000th inch thickness and manually fenestrated with slits at 0.5–1 cm intervals. Traction sutures were placed through the glans penis and used to stretch the penile shaft to its full length. The skin graft was inset to the penile shaft defect with a combination of skin staples, resorbable sutures, and tissue glue (ARTISS, Baxter Healthcare, USA).

**Figure 2:**
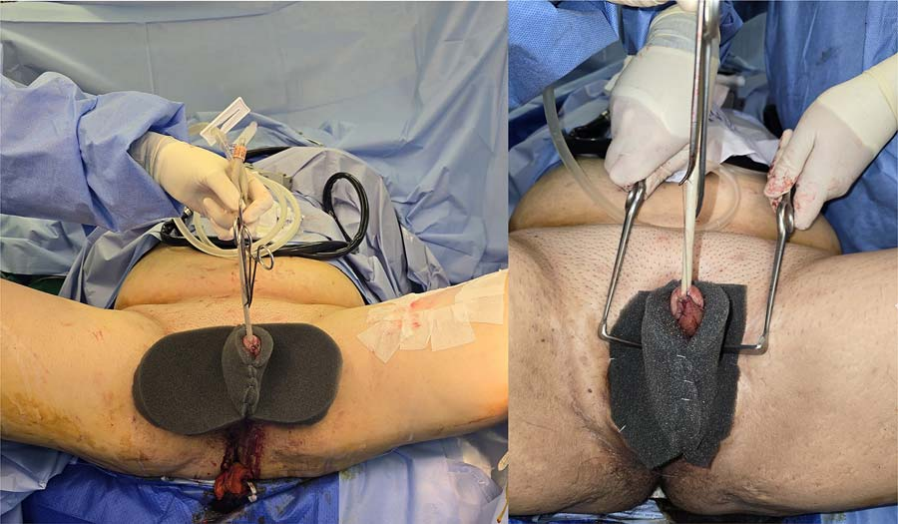
Application of double-opposing negative pressure dressing to splint the penile shaft.

Negative pressure dressing (V.A.C.® Therapy, Kinetic Concepts, Inc, USA) was applied in a double-opposing fashion as follows: the black sponge was split into half along its width, and the two halves were then ‘sandwiched’ against each other with the penile shaft at stretch in the middle. The excess foam was trimmed to size and secured with skin staples (Fig. 2). A non-adherent petrolatum dressing (UrgoTul, Urgo Medical, France) was used as an interface between the skin graft and the VAC (Vacuum-Assisted Closure, V.A.C.® Therapy, Kinetic Concepts, Inc, USA) foam, to prevent shearing of the skin graft upon dressing removal. A seal was achieved by sandwiching the black foam and adjacent segment of urinary catheter within two sheets of transparent VAC drapes of equal sizes. The negative pressure was set to 50 mmHg, continuous mode.

**Figure 3:**
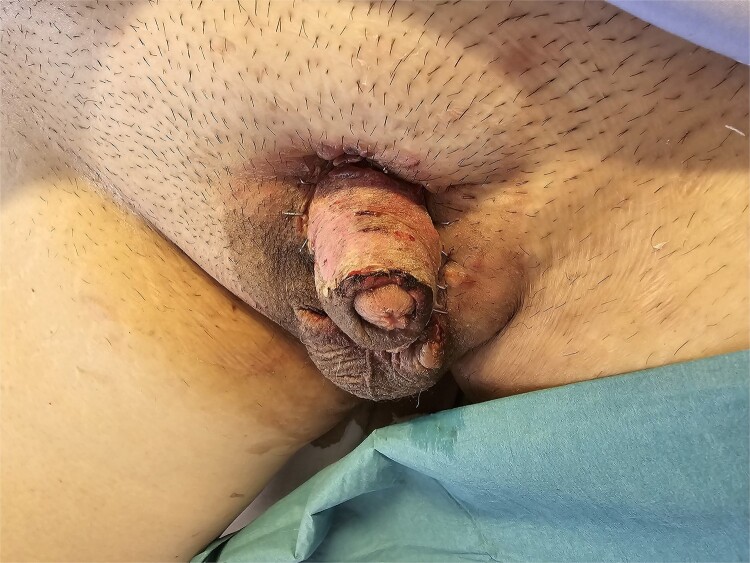
Good skin graft take and contour following 1 week of negative pressure dressing.

Post-operatively, negative pressure dressing was continued for a week, and the wounds were inspected on the seventh post-operative day. Good skin graft take was noted with no graft loss, adherence to the wound bed, and good conformation to the native penile shaft contour (Fig. 3).

## Discussion

The use of negative pressure dressings has been demonstrated to promote wound healing in animal and human studies [8, 9], and promote skin graft take as compared with conventional dressings [10, 11]. A less commonly described application is of its use in the penile shaft region, which is limited to a few case series [12, 13]. The proposed benefits of using negative pressure dressings are the increase in sponge stiffness and gentle compressive force at the sponge-tissue interface, which helps to splint the penile shaft [12]. We find this to be accurate and advantageous in our experience as well.

Weinfield et al. reported that the application of circumferential negative pressure dressing around the penile shaft is safe, with adequate glans perfusion demonstrated on post-operative capillary refill checks [12]. Their technique of dressing involves the application of multiple concentric ring-shaped pieces of sponge stacked sequentially on top of each other and secured with staples. In contrast, our technique involves the application of two opposing pieces of sponge sandwiched against each other. We find this to be advantageous for two reasons: first, it allows for more direct splinting of the penile shaft in its stretched and straight position as the sponges are in full contact and parallel to the vector of the penis; second, it is more straightforward to fashion, requiring only two equal pieces of sponge obtained by splitting it longitudinally. Nonetheless, this case report only represents our early experience with the technique, and serves as a technical description. Greater patient numbers with longer follow-up durations would be ideal to demonstrate its clinical efficacy.

We believe that this technique is relatively simple to adopt, and may allow reconstructive surgeons to manage skin grafts on the penile shaft with greater ease and confidence of optimum graft take.

## References

[1] Patino G , Zheng MY, Breyer BN, et al. Skin grafting applications in urology. Rev Urol 2019;21:8–14. 31239824 PMC6585185

[2] Lippin Y , Shvoron A, Tsur H. A simple splinting device for skin grafts of the penis. Ann Plast Surg 1992;29:185–6. 10.1097/00000637-199208000-00018. 1530274

[3] Netscher DT , Marchi M, Wigoda P. A method for optimizing skin graft healing and outcome of wounds of the penile shaft and scrotum. Ann Plast Surg 1993;31:447–9. 10.1097/00000637-199311000-00011. 8285531

[4] Yano K , Kubo T, Takagi S, et al. Fixation for skin grafting of the penis with polyurethane foam. Plast Reconstr Surg 2002;109:818–9. 10.1097/00006534-200202000-00071. 11818881

[5] Jordan DJ , Hough M. Introducing the doughnut inspired compression dressing for penile shaft reconstruction. J Plast Reconstr Aesthet Surg 2016;69:e154–5. 10.1016/j.bjps.2016.03.022. 27151756

[6] Dunev VR . A practical method of dressing and immobilizing the penis after using split-thickness skin graft. Int Wound J 2024;21:e14467. 10.1111/iwj.14467. 37942545 PMC10898387

[7] Murugan T , Choon KWL, Ong XS, et al. A systematic review and meta-analysis of mapping biopsy for primary Extramammary Paget’s disease in reducing recurrence following surgical excision. Ann Surg Open 2023;4:e339. 10.1097/AS9.0000000000000339. 38144489 PMC10735084

[8] Morykwas MJ , Argenta LC, Shelton-Brown EI, et al. Vacuum-assisted closure: a new method for wound control and treatment: animal studies and basic foundation. Ann Plast Surg 1997; 38:553–62. 10.1097/00000637-199706000-00001. 9188970

[9] Argenta LC , Morykwas MJ. Vacuum-assisted closure: a new method for wound control and treatment: clinical experience. Ann Plast Surg 1997;38:563–76; discussion 577. 10.1097/00000637-199706000-00002. 9188971

[10] Blackburn JH , Boemi L, Hall WW, et al. Negative-pressure dressings as a bolster for skin grafts. Ann Plast Surg 1998;40:453–7. 10.1097/00000637-199805000-00001. 9600426

[11] Agrawal N . Comparative study of outcomes between application of negative pressure wound therapy to split skin graft versus split skin graft immobilized by traditional bolster dressing. Int Surg J 2023;10:1317–24. 10.18203/2349-2902.isj20232327.

[12] Weinfeld AB , Kelley P, Yuksel E, et al. Circumferential negative-pressure dressing (VAC) to bolster skin grafts in the reconstruction of the penile shaft and scrotum. Ann Plast Surg 2005;54:178–83. 10.1097/01.sap.0000143606.39693.3f. 15655470

[13] Stokes TH , Follmar KE, Silverstein AD, et al. Use of negative-pressure dressings and split-thickness skin grafts following penile shaft reduction and reduction scrotoplasty in the management of penoscrotal elephantiasis. Ann Plast Surg 2006;56:649–53. 10.1097/01.sap.0000202826.61782.c9. 16721079

